# Geographical Detector-based influence factors analysis for Echinococcosis prevalence in Tibet, China

**DOI:** 10.1371/journal.pntd.0009547

**Published:** 2021-07-12

**Authors:** Tian Ma, Dong Jiang, Mengmeng Hao, Peiwei Fan, Shize Zhang, Gongsang Quzhen, ChuiZhao Xue, Shuai Han, WeiPing Wu, Canjun Zheng, Fangyu Ding

**Affiliations:** 1 State Key Laboratory of Resources and Environmental Information System, Institute of Geographic Sciences and Natural Resources Research, Chinese Academy of Sciences, Beijing, China; 2 College of Resources and Environment, University of Chinese Academy of Sciences, Beijing, China; 3 Key Laboratory of Carrying Capacity Assessment for Resource and Environment, Ministry of Natural Resources of the People’s Republic of China, Beijing, China; 4 Department of Geological Engineering and Environment, China University of Mining and Technology, Beijing, China; 5 Computer Network Information Center, Chinese Academy of Sciences, Beijing, China; 6 Tibet Autonomous Region Center for Diseases Control and Prevention, Lhasa, Tibet Autonomous Region, China; 7 National Health Council Key Laboratory of Echinococcosis Prevention and Control, Lhasa, Tibet Autonomous Region, China; 8 National Institute of Parasitic Diseases, Chinese Center for Disease Control and Prevention, Chinese Center for Tropical Diseases Research, WHO Collaborating Centre for Tropical Diseases, National Center for International Research on Tropical Diseases, Ministry of Science and Technology, Key Laboratory of Parasite and Vector Biology, MOH, Shanghai, China; 9 Chinese Center for Disease Control and Prevention (China CDC), Beijing, China; Centre Hospitalier Universitaire de Besancon, FRANCE

## Abstract

Echinococcosis, caused by genus *Echinococcus*, is the most pathogenic zoonotic parasitic disease in the world. In Tibet of the People’s Republic of China, echinococcosis refers principally to two types of severe zoonosis, cystic echinococcosis (CE) and alveolar echinococcosis (AE), which place a serious burden on public health and economy in the local community. However, research on the spatial epidemiology of echinococcosis remains inadequate in Tibet, China. Based on the recorded human echinococcosis data, maps of the spatial distribution of human CE and AE prevalence in Tibet were produced at city level and county level respectively, which show that the prevalence of echinococcosis in northern and western Tibet was much higher than that in other regions. We employ a geographical detector to explore the influencing factors for causing CE and AE while sorting information on the maps of disease prevalence and environment factors (e.g. terrain, population, and yak population). The results of our analysis showed that biological factors have the most impact on the prevalence of echinococcosis, of which the yak population contributes the most for CE, while the dog population contributes the most for AE. In addition, the interaction between various factors, as we found out, might further explain the disease prevalence, which indicated that the echinococcosis prevalence is not simply affected by one single factor, but by multiple factors that are correlated with each other complicatedly. Our results will provide an important reference for the evaluation of the echinococcosis risk, control projects, and prevention programs in Tibet.

## Author summary

Echinococcosis, a worldwide zoonosis caused by genus Echinococcus, is highly endemic in Tibet of China. CE and AE as two main types of Echinococcosis are causing serious health burdens, thereby attracting more attention to public health in Tibet. This work provides detailed geographical information about CE and AE prevalence based on the recorded human echinococcosis data from CDC. A geographical detector was employed to explore the potential environmental factors that influence these diseases and to quantify the specific contribution of each factor as well as discovering the interaction between factors. The results showed that biological factors have the most impact on the prevalence of echinococcosis, of which the yak population contributes most to CE while the dog population makes the most contribution to AE. Besides, the interactive results greatly improved the explanation of the two diseases, indicating that the echinococcosis prevalence is not simply affected by one factor, but by various interrelated factors. Such research can be beneficial to the evaluation, control projects, and prevention programs of the echinococcosis risk in Tibet.

## Introduction

Echinococcosis is considered to be the most pathogenic zoonosis caused by cestode species of the genus *Echinococcus* (family *Taeniidae*) in the adult or larval stages [1, 2]. Transmission of echinococcosis depends on carnivores as definitive hosts and various other animals as intermediate hosts. Human beings as accidental intermediate hosts can be infected with echinococcosis by directly ingesting eggs of *Echinococcus spp*. or contacting with contaminated environment indirectly [3, 4]. Cystic echinococcosis (CE) and alveolar echinococcosis (AE), caused by the adult or larvae stage of *Echinococcus granulosus (E*.*granulosus*) and *Echinococcus multilocularis (E*.*multilocularis)* respectively, are the two main types of echinococcosis, which may result in asymptomatic infections that lead to severe and even fatal disease [1]. The asymptomatic period of CE can last several years(2–15 years)[3], until the echinococcal cyst grows to a certain stage when clinical symptoms start manifesting(e.g. liver pain, dizziness, abdominal distention). Its mortality rates range from 2% to 4%, but it may increase considerably without appropriate treatment [5]. Human AE, compared to CE, has a longer asymptomatic period (5–20 years). It is imperative to notice that its mortality rate in untreated or inappropriately treated patients exceeded 90% within 10 to 15 years [6]. Given serious medical, social, and economic consequences in those underpopulated regions, echinococcosis has been listed as one of the 17 neglected tropical diseases (NTDs) by the World Health Organization (WHO) awaiting control or elimination by 2050 [7].

Echinococcosis is a near-cosmopolitan zoonosis, which imposes a huge burden on public health management [8]. CE has a worldwide geographic range in association with herding. It is endemic in regions of Eurasia (e.g. Mediterranean region, Russian central Asia, and China), northern and eastern Africa, Australia, and South America. The current estimate of the global burden of CE is approximately 188,000 new cases per annum leading to 184,000 disability-adjusted life years (DALYs) [9], out of which 40% DALYs were in China. Additionally, the annual loss was estimated to be US$ 3 billion due to the treatment costs, lost wages, and livestock-associated production losses, of which China holds an important share [9]. AE is considered prevalent in temperate and arctic regions of the Northern Hemisphere, mainly distributed in regions of Alaska, northern and central Europe, central Asia, and China [10]. According to the estimate, approximately 18,235 new AE cases resulting in 666,434 DALYs per annum globally, of which about 1600 cases and 33,000 DALYs occurred in Europe, Russia, and Central Asia, while about 91% of the new cases and 95% of the DALYs were estimated to be in China [11–13].

**Fig 1:**
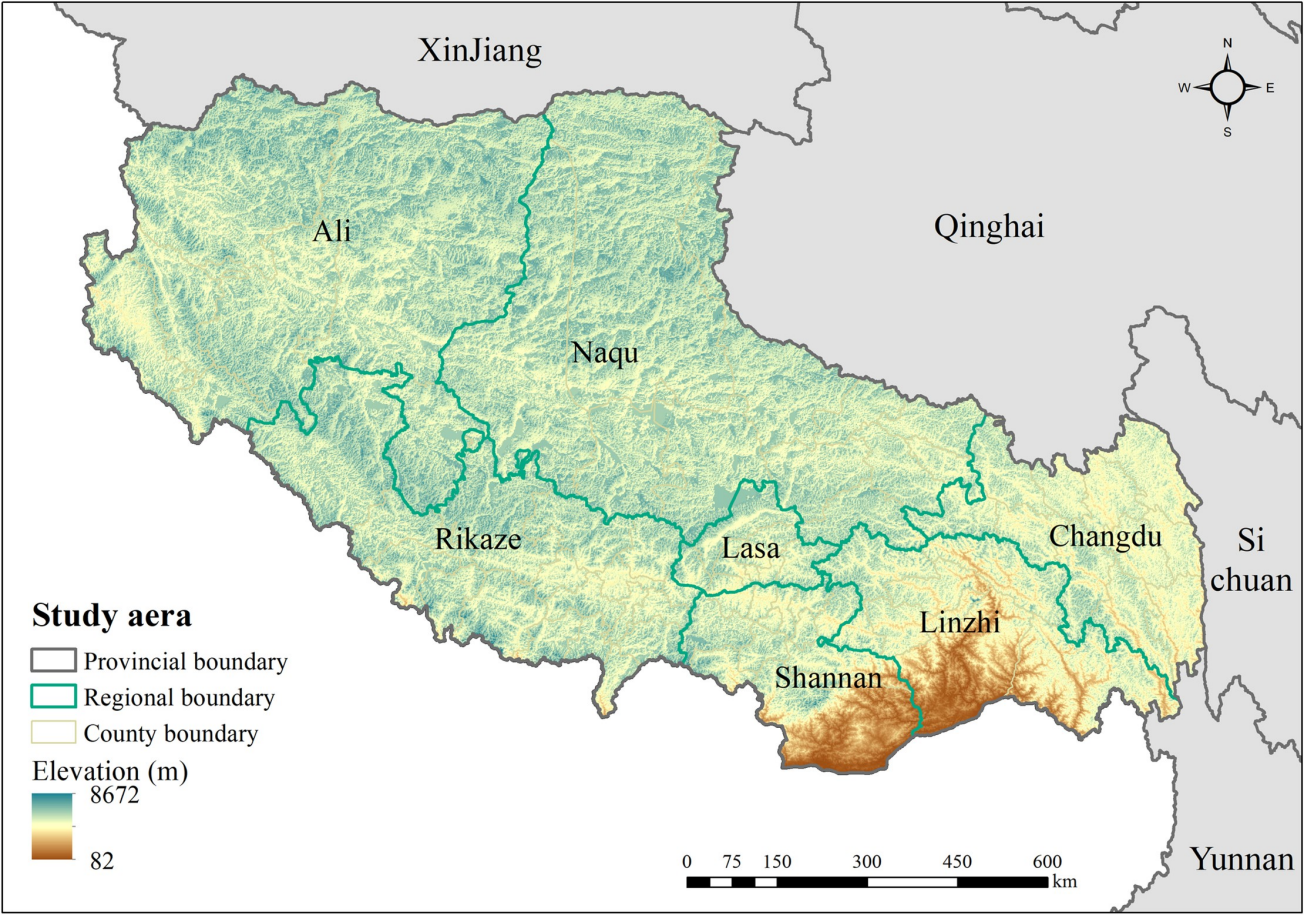
Study area—The Tibet Autonomous Region (Tibet) of the People’s Republic of China. Source of map: https://www.worldofmaps.net/typo3temp/images/chinakarte.jpg.

China as one of the most important and highly endemic regions for both CE and AE holds a high percentage of the global burden of echinococcosis [3]. It is found that CE is endemic in at least 368 counties in China, out of which 119 are subject to AE as well, while most of them are located in western and north-western China, including regions of Gansu, Qinghai, Sichuan, Ningxia Hui Autonomous, Xinjiang Uygur Autonomous, and the Tibet Autonomous [3, 11]. A national epidemiological survey conducted between 2012 and 2016 showed that there are estimated to be 50 million people at risk of contracting echinococcosis in western and north-western China, of whom about 170,000 people are cases with echinococcosis. The survey furthermore illustrated that the prevalence of human echinococcosis in Tibet was 1.66%, which was much higher than the average prevalence of echinococcosis in other parts of China (0.24%)[14], thusly seriously demonstrated the threat to the health of local people and the limitation of the economic development in Tibet. Despite this situation, research on echinococcosis in China remains greatly inadequate compared to that in other countries [15]. Meanwhile, previous literatures have showed that the spatial distribution of echinococcosis was linked to geographical, meteorological, biological and socio-economic factors [16–24], while the main drive factors of echinococcosis has not been comprehensively and systematically analysed. Thus, in this study, we select the Tibet Autonomous as the study area (Fig 1), attempting to map the spatial distribution patterns of CE and AE and to explore the contribution of the potential environmental risk factors of the diseases, which is critical for decision-making that facilitates the design, implementation, and monitoring of spatially targeted interventions to reduce the burden of human echinococcosis in Tibet.

## Material and method

### Ethics statement

This study was approved by the Ethics Committee of CDC. All the data analyzed in this study were anonymized to protect patient confidentiality.

### Study area

The Tibet Autonomous Region (Tibet) is located in the southwest of the People’s Republic of China, bordering provinces of Xinjiang, Sichuan, Qinghai, and Yunnan (Fig 1), stretches from 26°50′N to 36°53′N and from 78°25′E to 99°26′E, with an average altitude of more than 4000 meters. Administratively, Tibet is divided into 7 cities (regions), which are further subdivided into 74 counties, with a permanent population of 3.17 million and a total area of 1.2 million square kilometres.

### Disease dataset

Echinococcosis data used in this study was obtained from the Chinese Center for Disease and Prevention (CDC), which was derived from an epidemiological survey in Tibet between 2012 and 2016. A stratified and proportionate sampling method was employed to select samples in 380 villages of 74 counties in Tibet, and a total of 80384 residents were selected. Among them, 1202 were examined positive for CE while 153 were tested positive for AE by B-ultrasonography diagnostic. The overall human prevalence of CE and AE in Tibet were 1.5% (1202/80384) and 0.19% (153/80384) respectively. Detailed information including age, sex, occupation, etc is also included in the disease data. Data used in this study had been de-identified to protect patient confidentiality. Other detailed information about the disease data can be found elsewhere [14].

### Determinants of Echinococcosis and their proxies

**Fig 2:**
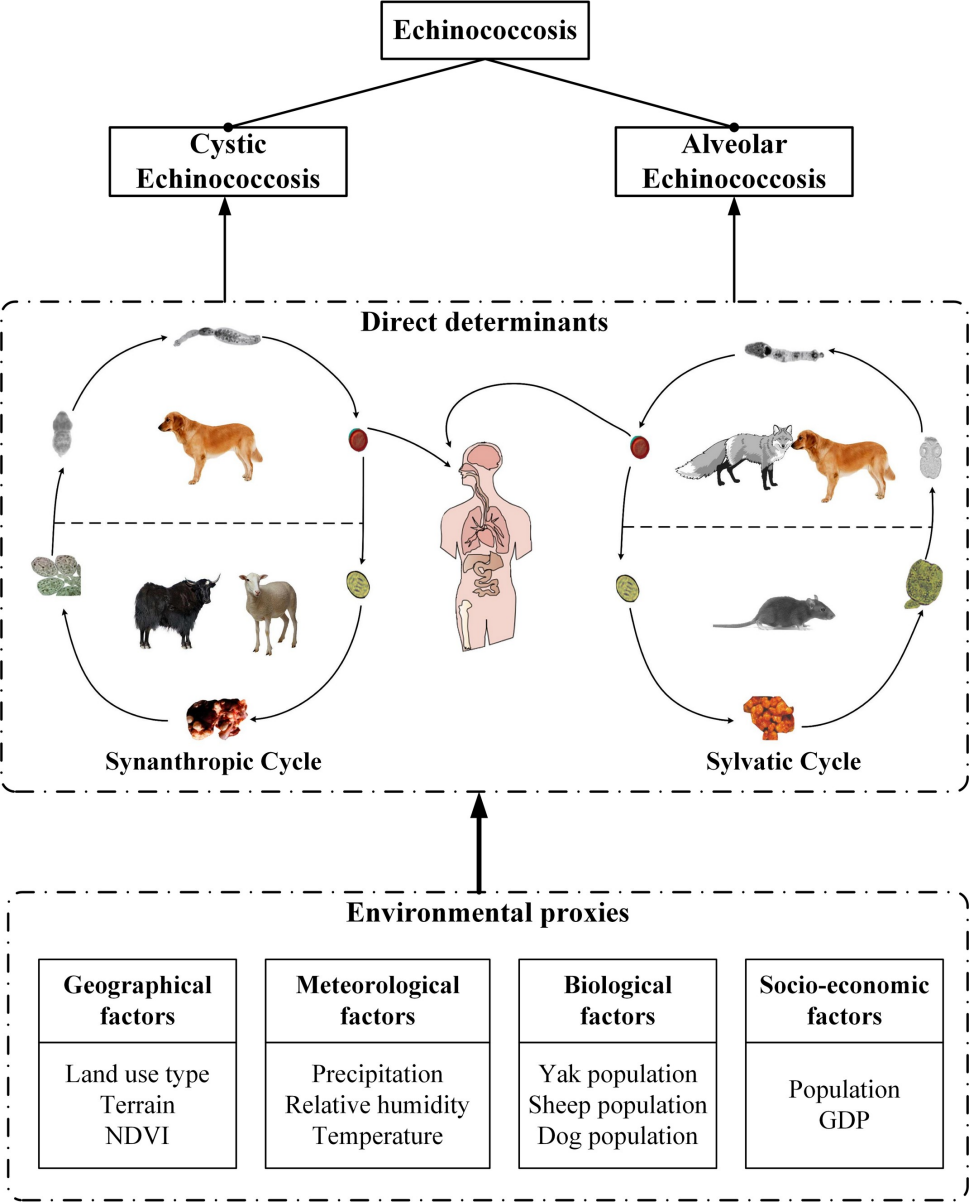
Direct determinants and environmental proxies. Note: Symbols of fox and rat in “Direct determinants” section were shown in grey due to data missing.

Fig 2 illustrated a conceptual framework of direct determinants and environmental proxies for CE and AE. The key determinant leading to human CE and AE was the accidental exposure to *E*.*granulosus* and *E*.*multilocularis* respectively during the entire life cycle of echinococcosis transmission. The transmission of CE and AE relies on both the sylvatic cycle and the synanthropic cycle. CE primarily maintained in the synanthropic cycle, which involved livestock such as cattle (yak) and sheep that are regarded as the most important intermediate reservoir hosts while canines (mainly domestic dogs) are the main ultimate hosts in Tibet [25]. For AE, in the most endemic regions of the world (e.g. Europe, Japan, and North America) where foxes, wolves are the main definitive hosts, the sylvatic cycle is reported as the main transmission pattern of *E*. *multilocularis* [7, 10]. In China, however, the synanthropic cycle in which dogs are the predominant definitive hosts seems to be the main transmission pattern and is responsible for human infections of AE [26]. Humans usually are infected with echinococcosis as accidental intermediate hosts. Though pathogens of diseases are too difficult to observe directly, environmental factors that particularly influence human behaviour, animal population, the spatial distribution of intermediate and definitive hosts, and the survival of the parasite eggs, are important for the successful transmission of echinococcosis and thus may contribute to the risk of human exposure as well as catching CE or AE [27, 28]. Meanwhile, those influencing factors are measurable in geographical space. Therefore, it is possible to explore the potential risk factors of the diseases by investigating their environmental proxies. Specific environmental proxies are listed as follows:

### Geographical factors

**Fig 3:**
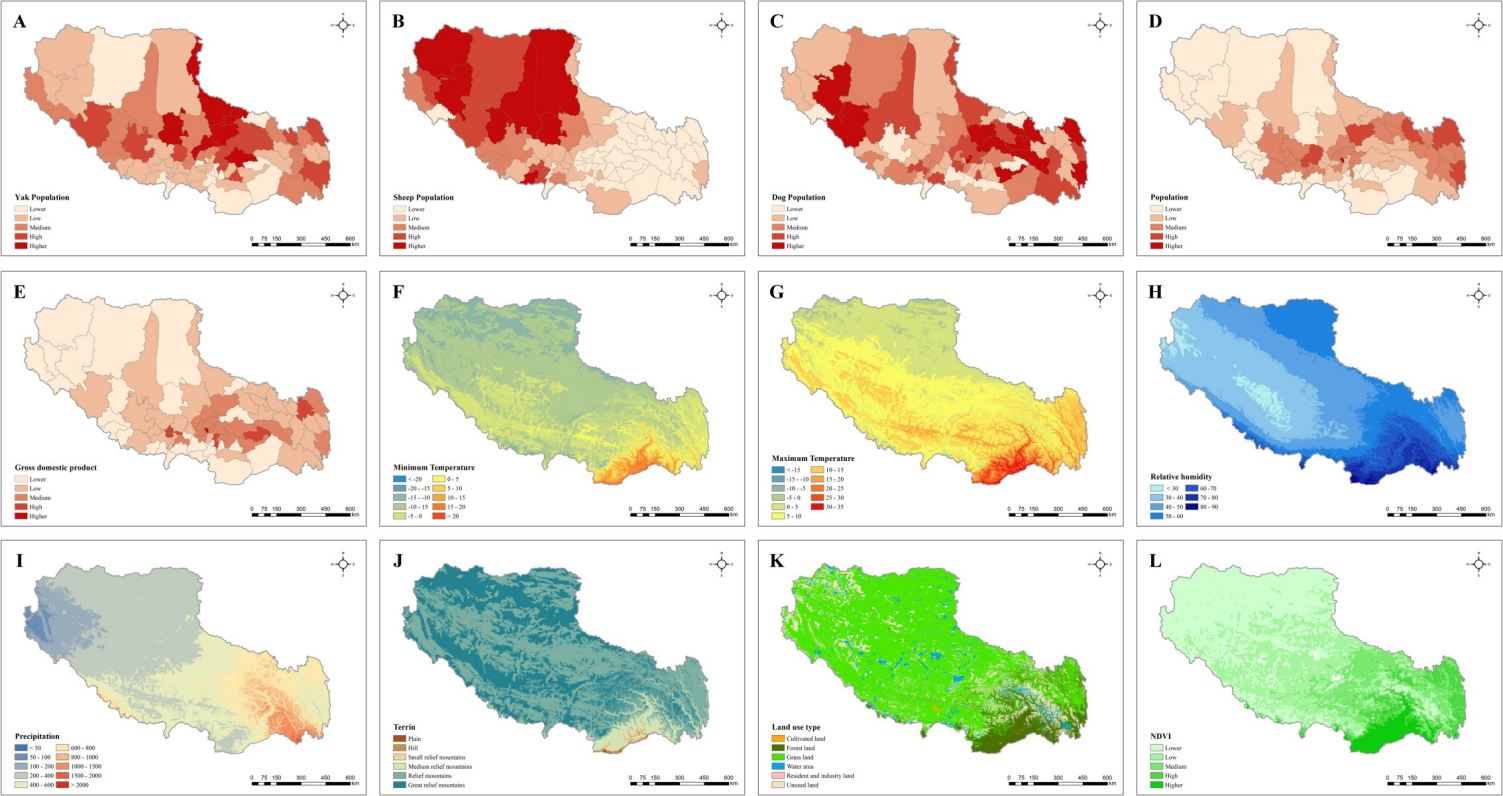
Spatial distribution of environmental proxies. Note: A = Yak population, B = Sheep population, C = Dog population, D = Population, E = GDP, F = Minimum temperature, G = Maximum temperature, H = Relative humidity, I = Precipitation, J = Terrain, K = Land use type, L = NDVI. Abbreviations: GDP, gross domestic product; NDVI, normalized difference vegetation index. Source of map: https://www.worldofmaps.net/typo3temp/images/chinakarte.jpg.

High altitude grasslands on the Tibetan Plateau enable pastoralism to flourish, which is associated with recognized risks of human echinococcosis for livestock and dogs. Echinococcosis transmission can occur in different landscape types where various physical and biological factors combine to determine the intensity of the parasite transmission [20, 21]. On the other hand, Land-use type and normalized difference vegetation index (NDVI) are important limitations to the distribution of echinococcosis hosts [22, 23]. Hence, we take land-use type, NDVI, and terrain as geographical proxies, downloaded from the Data Center for Resources and Environmental Sciences, Chinese Academy of Science (RESDC) (http://www.resdc.cn) and shown in Fig 3J, 3K and 3L.

### Meteorological factors

Some previous studies have found that the activity and survival time of *E*.*granulosus* and *E*. *multilocularis* eggs were sensitive to fluctuation in both temperature and humidity [29, 30]. More than that, the abundance and distribution of host species are affected by climate change, and therefore increase the potential risk of human exposure to echinococcosis. Thus, in this study, precipitation, relative humidity, and temperature were selected as meteorological proxies. The daily climate data spanned 2005 to 2016 was available on the China Meteorological Data Service Center (http://data.cma.cn), based on which annual average precipitation, annual average minimum (maximum) temperature, and annual average relative humidity were obtained on ANUSPLIN-SPLINA software. Classified maps of minimum (maximum) temperature, relative humidity, and precipitation were pictured in Fig 3F, 3G, 3H and 3I respectively.

### Biological factors

As the most important domestic reservoir hosts, yak, and sheep that are susceptible to echinococcosis in Tibet, play important roles in the transmission of human echinococcosis [3, 25]. In addition, dogs on the Tibetan Plateau are important definitive hosts for both *E*.*granulosus* and *E*.*multilocularis* [31, 32]. The population of host animals will affect the prevalence of echinococcosis among animals, while the proximity of humans to animal hosts (definitive and intermediate hosts) will increase the potential risk of human exposure to echinococcosis, thus directly affect the prevalence of human echinococcosis. We take yak, sheep, and dog populations, obtained from the epidemiological survey of CDC, as biological proxies in our study. Classified maps of yak, sheep, and dog populations were pictured in Fig 3A, 3B and 3C respectively.

### Socio-economic factors

Some studies have found that socioeconomic factors are associated with the prevalence of human echinococcosis [20, 22, 23], for they deeply affect people’s lifestyles, traditional living habits, education levels, and local health conditions. In this study, since the population and gross domestic product (GDP) could in some degree reflect the level of regional economic development, we consider population and GDP as factors of socio-economic proxies, which can be downloaded from the Data Center for Resources and Environmental Sciences, Chinese Academy of Science (RESDC) (http://www.resdc.cn). Classified maps of population and GDP were shown in Fig 3D and 3E respectively.

### Geographical detector

The geographical detector was proposed by Wang et al in 2010 [33], which is a statistical tool based on the spatial stratified heterogeneity of geographical variables to explore the environmental risks of interesting variables. In this study, it was used to explore environmental risks to the prevalence of CE and AE in Tibet, that is, to compare the spatial consistency of disease prevalence with the geographical strata (e.g., land use, terrain, NDVI, precipitation, humidity, temperature, biological population, GDP, etc) in which potential risk determinants exist [34].

**Fig 4:**
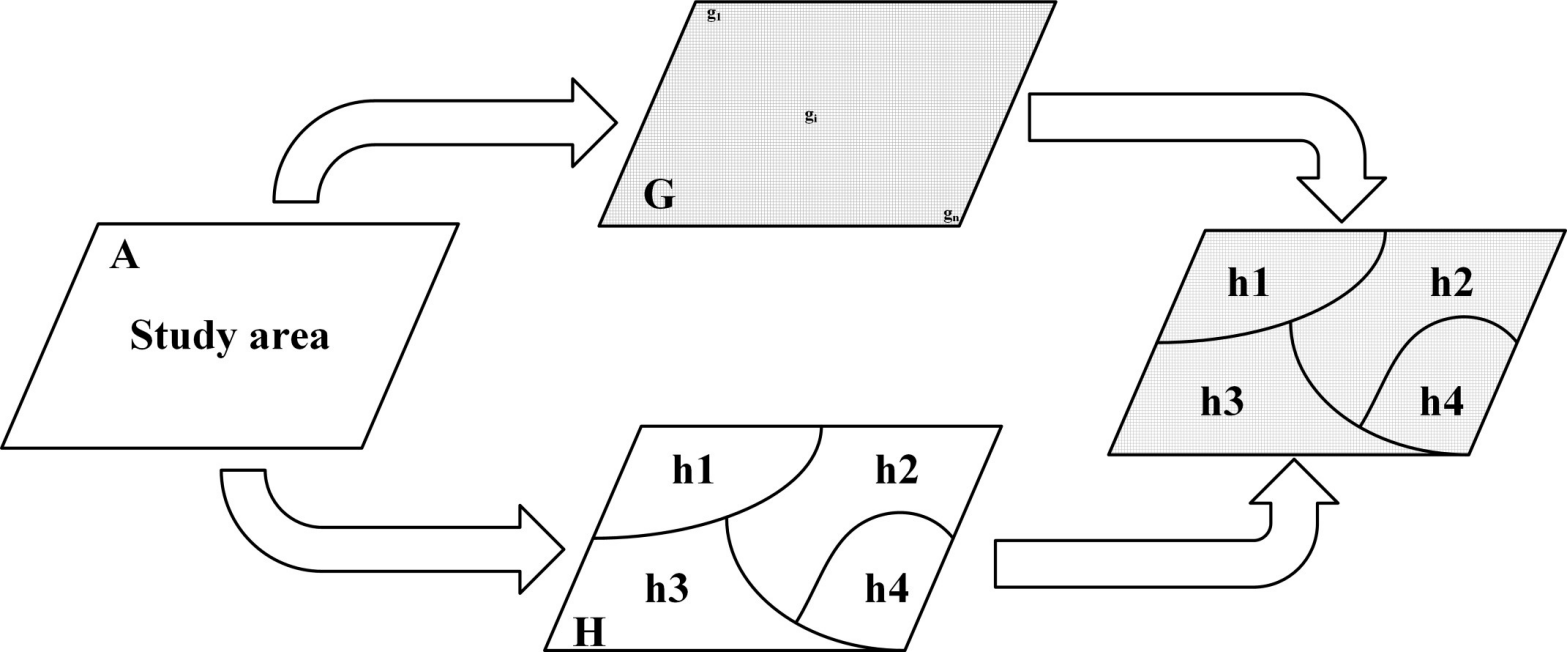
Schematic diagram of the geographical detector. A Study region, G grid system, H geographical stratum.

Fig 4 depicts the concept and diagram of the geographical detector. We consider a study area A with disease prevalence (e.g. CE and AE) recorded on a grid system G, which is overlaid by potential risk layers H (e.g. yak and dog population), denoted as H = {H_i_}. The mean value and variance of disease prevalence over sub-regions of layer H are denoted as $\bar{y_{h,i}}$ and $\delta_{h,i}^{2}$ respectively. The mechanism is measured by the value of q-statistic, which is defined by equal 1:

$$q=1-\frac{\sum_{h=1}^{L}N_{h}\delta_{h}^{2}}{N\delta^{2}}$$ (1)

Where the CE and AE prevalence were composed of N units. L denotes the number of strata. N_h_ and $\delta_{h}^{2}$ express the size and variance of stratum h. The value of q-statistic ranges from 0 to 1. On one hand, if disease prevalence is completely controlled by factor H, the q value shall equal 1. On the other hand, if disease prevalence and factor H are not related, the q value equals 0 [35]. Meanwhile, it can also represent the extent to which factor H interprets the disease prevalence. The larger the q-value, the greater the impact of the factor.

The geographical detector is composed of risk detector, factor detector, ecological detector, and interaction detector. The risk detector indicates where the risk areas are. The factor detector quantifies the impact of an environmental factor on the risk of disease prevalence. And the ecological detector explores whether stratum A is more significant than stratum B in controlling the spatial distribution of disease prevalence. The interaction detector reveals the interactive effect of two factors on the disease prevalence. Table 1 shows the description of interactive results in which *q*(*X*1∩*X*2) refers to the interaction of factor X1 and X2. In this study, the package “geo-detector” in R version 3.5.2 (64-bite version) was used to employ the geo-detector method, which can be downloaded from the website of http://geodetector.cn/. The website also provided the excel version enclosed examples of datasets. More detailed information about the geographical detector method can be found elsewhere [33, 34].

**Table 1:** Description of interactive results.

Description	Interaction
q(X1∩X2)<Min(q(X1),q(X2))	Weaken, non-linear
Min(q(X1),q(X2))<q(X1∩X2)<Max(q(X1),q(X2))	Weaken, univariate
q(X1∩X2)>Max(q(X1),q(X2))	Enhance, bivariate
q(X1∩X2)=q(X1)+q(X2)	Independent
q(X1∩X2)>Max(q(X1),q(X2))	Enhance, non-linear

## Results

### Spatial distribution of Human Echinococcosis prevalence in Tibet

**Fig 5:**
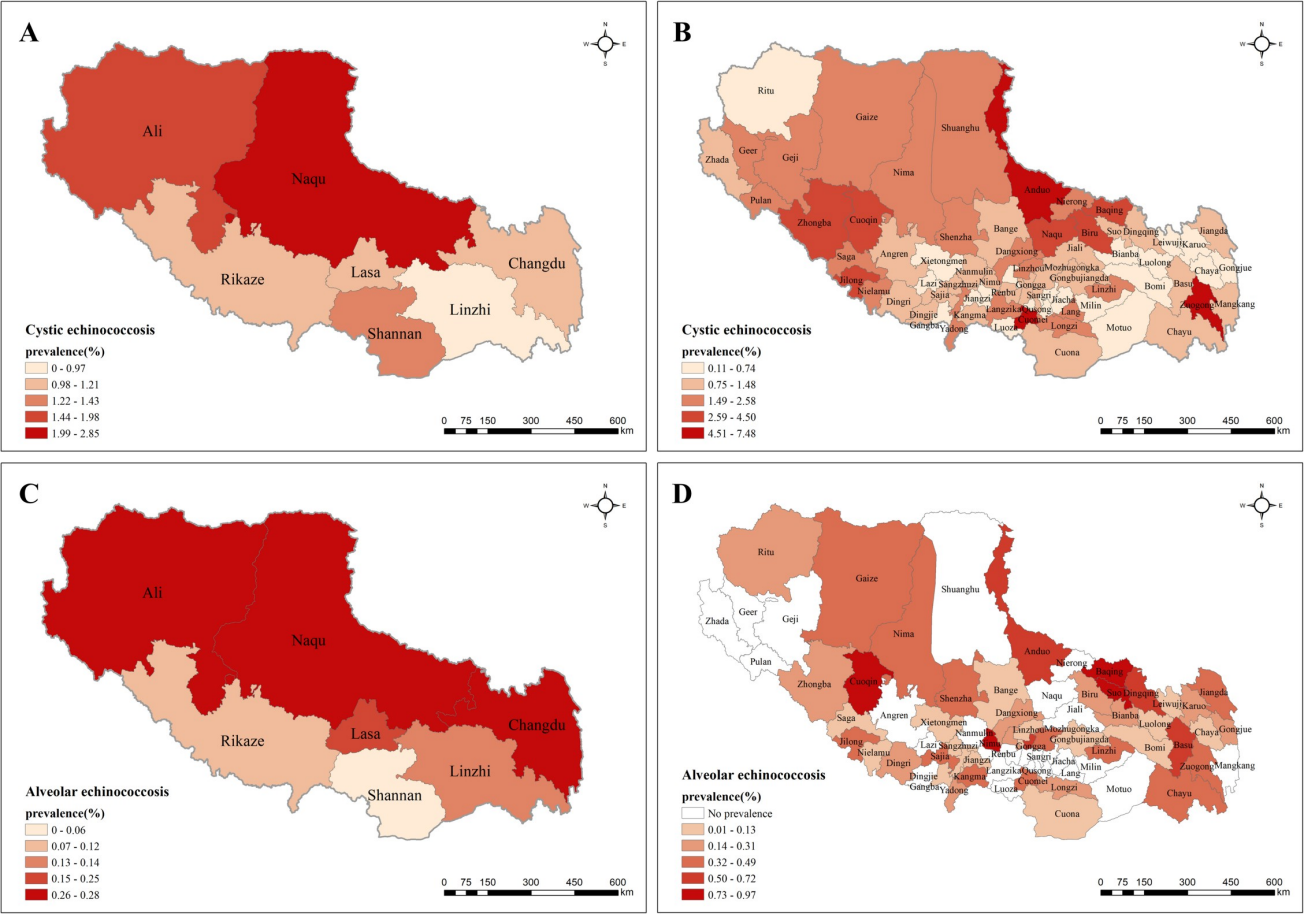
Spatial distribution patterns of Echinococcosis prevalence in Tibet. (A) CE prevalence at city level; (B) CE prevalence at county level; (C) AE prevalence at city level; (D) AE prevalence at county level. Source of map: https://www.worldofmaps.net/typo3temp/images/chinakarte.jpg.

The prevalences of human echinococcosis at different levels in Tibet are presented in Fig 5. Fig 5A and 5C show the spatial distribution of prevalence for CE and AE at city level respectively (details listed in Table 2) while Fig 5B and 5D depict the prevalence of CE and AE at county level. The ten counties with the highest prevalence were shown in Table 3.

**Table 2:** Prevalence at city level of CE and AE in Tibet.

	City/Region	No.of positive/total	Prevalence (%)		City/Region	No.of positive/total	Prevalence (%)
**CE**	Naqu	339/11897	2.85	**AE**	Naqu	33/11897	0.28
	Ali	94/4740	1.98		Ali	13/4740	0.27
	Shannan	160/11184	1.43		Changdu	39/14289	0.27
	Rikaze	261/21497	1.21		Lasa	27/10917	0.25
	Changdu	167/14289	1.17		Linzhi	8/5860	0.14
	Lasa	124/10917	1.14		Rikaze	26/21497	0.12
	Linzhi	57/5860	0.97		Shannan	7/11184	0.06

**Table 3:** Top 10 counties of Tibet in terms of prevalence for CE and AE.

City /Region		county	No.of positive/total	Prevalence(%)	City /Region		county	No.of positive/total	Prevalence(%)
**CE**	Changdu	Zuogong	66/882	7.48	**AE**	Naqu	Baqing	8/823	0.97
	Shannan	Cuomei	60/897	6.69		Ali	Cuoqin	8/824	0.97
	Naqu	Anduo	44/833	5.28		Naqu	Suo	7/812	0.86
	Naqu	Baqing	37/823	4.50		Lasa	Nimu	7/853	0.82
	Rikaze	Zhongba	31/819	3.78		Naqu	Ando	6/833	0.72
	Naqu	Naqu	115/3312	3.47		Changdu	Dingqing	10/1625	0.61
	Naqu	Biru	48/1612	2.98		Changdu	Basu	5/813	0.61
	Rikaze	Jilong	36/1216	2.96		Lasa	Dazi	5/834	0.60
	Ali	Cuoqin	23/824	2.79		Lasa	Mozhugongka	4/820	0.49
	Naqu	Nierong	21/813	2.58		Rikaze	Kangma	4/855	0.47

The spatial distribution of human CE prevalence at the city level was shown in Fig 5A, which reveals that the high prevalence areas are mainly concentrated in northern and western Tibet, whereas prevalence in south-eastern Tibet was relatively low. For example, the highest prevalence was in Naqu region with a value of 2.85% (339/11897), followed by which in Ali region with a value of 1.98% (94/4740). By contrast, the prevalence in Linzhi region was less than 1%, with a value of 0.97% (57/5860). Fig 5B pictures detailed CE prevalence at the county level, indicating that prevalence in northern Tibet was higher than that in southern Tibet in general, which was corresponding to its distribution of city level. Besides, it is worth noticing that there is spatial heterogeneity in the prevalence of each city geographically. For instance, the prevalence in eastern Naqu, northern Rikaze, and southern Changdu was significantly higher than that in other parts of each city/region. In addition, it should be strongly noted that there were two counties with remarkably high prevalence, namely, Zuogong county of Changdu region with 7.48% (66/882) and Cuomei county of Shannan region with 6.69% (60/897), whereas the prevalence of their adjacent areas were only around 1%.

The human AE prevalence at the city level based on geographical distribution was depicted in Fig 5C, which demonstrates that the prevalence in northern regions including Ali, Naqu, and Changdu was much higher than that in southern regions (Rikaze, Shannan, and Linzhi). The maximum prevalence was 0.28% (33/11897) in Naqu, followed by Ali (13/4740) and Changdu (39/14289) with a value of 0.27%, whereas the minimum prevalence was only 0.06% (7/11184) in Shannan region. Fig 5D describes the prevalence of AE at county level, from which we could conclude that there were 27 counties (36%) in Tibet spared from AE. The ten counties with the highest prevalence were scattered in regions of Tibet. Among them, Baqing county of Naqu region (8/823) and Cuoqin county of Ali region (8/824) had the maximum prevalence with a value of 0.97%, followed by Suo county, Ali region of 0.86% (7/812) and Nimu county, Lasa of 0.82% (7/853).

### Influence factors analysis of Human Echinococcosis prevalence

The q-statistic listed in S1 Table was calculated by the factor detector of the geographical detector method. The factor detector reveals the influence of environmental proxies on the prevalence of human echinococcosis, including geographical, meteorological, biological, and socioeconomic factors. The value of q-statistic represents the contribution of related factors for the disease prevalence. For human CE, the yak population and sheep population make the greatest contribution with the q-statistic value of 0.26 and 0.16 respectively, whereas other values of factors are less than 0.1. For human AE, the dog population contributes the most (0.37), followed by the yak population (0.22), while other factors only play a small part in the prevalence of AE, to which the P values of factor detector correspond were listed in S2 Table.

The interactive q-statistic values of related factors for CE and AE were presented in S3 and S4 Tables, calculated by the interaction detector of the geographical detector, probing the joint impacts of two factors (for example: yak and dog population) on the disease prevalence. For CE, though the q-value for yak, sheep, and dog population were 0.26, 0.16, and 0.06 respectively, the interactive value of yak and sheep population was up to 0.59, and that value of yak and dog population was 0.45, sheep and dog population was 0.52, which further completed the interpretation of CE prevalence. In addition, we found that the interaction with the yak population will significantly amplify the ultimate effect upon the disease prevalence. For instance, the value of interactive effects between yak population and GDP was 0.49, and that with minimum temperature was 0.4, maximum temperature (0.42), relative humidity (0.39), precipitation (0.42), and NDVI (0.42). Furthermore, even for those factors doing little contribution, the interactions between them could noticeably enhance their separate effects on CE prevalence. For example, the q values of GDP and minimum temperature were 0.05 and 0.06 respectively, but their interactive value was 0.17.

Same with AE, the q-value for yak, sheep, and dog population were 0.22, 0.06, and 0.37 respectively while the interactive value of yak and sheep population was 0.52, and that of yak and dog population was 0.55, of sheep and dog population was 0.58, all of which greatly improved the explanation for the prevalence of AE. Besides, we found that when any factor interacts with the dog population, the interactive q-statistic value would increase surprisingly. For instance, the interactive effects value of dog population and population was 0.57, GDP (0.57), minimum temperature (0.47), maximum temperature (0.47), relative humidity (0.49), precipitation (0.50), terrain (0.43), Land use (0.44) and NDVI (0.49). Furthermore, even of those factors with little impact, interactions between them would enhance their separate effects on AE prevalence. For example, the q values of the sheep population and population were 0.06 and 0.05 respectively, but their interactive value was 0.38. Almost all the interactive values listed in S3 and S4 Tables seem to be higher than the value of any single factor (S1 Table), according to the description of Table 1, the interaction of different factors results in binary enhancement or non-linear enhancement to the prevalence of CE and AE.

## Discussion

As one kind of neglected zoonosis, echinococcosis seriously endangers human health and imposes a heavy burden on many countries due to the considerable morbidity rates and high economic losses. CE has a wider geographical distribution and a higher prevalence rate compared with AE, whereas the global burden of AE (666,434 DALYs) was much higher than CE (184,000 DALYs). The much lower human health burden of CE compared with AE was almost entirely due to the low mortality rate of CE relative to AE [9, 12]. However, it is important to note that as the lifecycle of CE in many countries involves livestock as intermediate hosts, there can be severer economic and animal health repercussions than that of AE, and the losses in the livestock industry have been estimated to be possibly up to US $2 billion annually. Due to the highest CE and AE prevalence of the world were basically concentrated in Tibet, it is of great significance to explore the spatial distribution characteristics and to study the potential risk factors of the diseases, which could provide local hydatid prevention and control strategies, thereby reducing the burden of the diseases.

Based on the data derived from an epidemiological survey of CDC in 2012 and 2016, the spatial distribution patterns of CE and AE prevalence in Tibet were mapped at different geographical scales. Maps at city level indicated that both CE and AE regions of northern Tibet (especially Naqu and Ali region) are high endemic areas, which may be because that these areas are mainly dominated by animal husbandry. Also, the county-level maps showed that CE was prevalent in all counties of Tibet while AE occurred in about 64% of counties, which also underlines counties of Anduo, Baqing, Cuoqin, Cuomei, and Zuogong presenting high co-endemicity of CE and AE, therefore, it can provide important information for the government on regionalization of key prevention and control efforts.

In addition, the geographical detector method was used to explore the risk factors for CE and AE in this study. The results showed that biological factors (yak, sheep, and dog population) influence the most in the prevalence of echinococcosis. For CE, it is perhaps a little surprising that there was no such a direct association strongly related to dogs, whereas the dogs are the obligatory definitive host of this parasite. However, there is an interaction between dogs and yaks or sheep (AB&C in S3 Table), which may be because CE requires both dogs and a suitable livestock intermediate host for transmission, hence it manifest a much stronger interaction when these intermediate hosts are present. For AE, we found that it is strongly associated with dog populations, which supports recent evidence that dogs are a major infection source of AE for humans[36]. It is also worth noting that periodic treatment of dogs with anthelmintic has reduced the prevalence of *E*.*multilocularis* in dogs in Western Sichuan province, along with a reduction in human AE. However, there is no change in the prevalence in small mammals[37], which illustrates the independent wild life cycle between foxes and small mammals that is not disturbed by the treatment of dogs. In daily life, due to the proximity of livestock and dogs, herdsmen are more susceptible to echinococcosis infection than others. Hence, deworming canine and improved slaughterhouse management (e.g. offal destruction and preventing dogs from feeding on infected organs of ungulates) as well as vaccination of livestock can be efficient intervention to control and prevent the spread of echinococcosis [19, 38]. In addition, strengthening local health education (especially for herdsmen) and organizing regular health examinations (e.g. ultrasound scanner and CT) for local herdsmen are necessary measures to detecting echinococcosis timely and to protect people’s lives as well as reducing the burden of the diseases.

Furthermore, it should be noted that although other factors (geographical, meteorological, and socio-economic factors) contribute little to the prevalence of echinococcosis based on factor detector, the interaction between them and those biological factors can significantly increase the interpretability of disease prevalence (e.g. the first row in S3 Table and the third row in S4 Table), which indicated that the echinococcosis prevalence was not affected simply by one single factor but by multiple factors with complexity. For instance, the interactive q-statistic value of the yak population with the maximum temperature (0.07) and precipitation (0.06) was 0.42 for CE. Meanwhile, the interactive q-statistic value of dog population with the maximum temperature (0.02) and precipitation (0.06) were 0.47, and 0.5 respectively for AE, which showed that the interpretability for the diseases was greatly enhanced when temperature and precipitation interacted with animal population. It is probably because that temperature and precipitation were important limitations to the activity and survival time of Echinococcosis eggs in animal hosts [20, 28, 30, 39], thus affecting the whole transmission cycle. In addition, Franz et.al have found that dog ownership and low income are potential risk factors associated with AE through systematic review and meta-analysis [22]. Also, Possenti et.al discovered that living in rural areas or with low income were significantly associated with CE derived from case-control studies[23], which could provide theoretical support for our results that the interactive q-statistic value of GDP and yak population was 0.49 for CE and the interactive q-statistic value of GDP and dog population was 0.57 for AE.

In the present study, we also conducted a risk detector. Risk detection may imply where the risk areas are. According to factor detection and interaction detection, biological factors have the greatest impact on echinococcosis. Thus, we focus on the risk detection results of biological factors, which were listed in S5 Table. It uncovers that the values of the average prevalence of CE in regions of yak population (from level 2 to 5) are 1.24%, 1.61%, 2.25% and 3.33%, while in regions of dog population (from level 1 to 4) are 1.38%, 1.49%, 1.75% and 2.29%, indicating that higher CE prevalence might occur in regions with higher yak and dog population. In addition, it should be noted that the average prevalence of CE in regions of the yak population at level 1 was higher than that at level 3, which may due to the maximum sheep population occurred in regions with the minimum yak population (Fig 3). Meanwhile, we found that the average prevalence of CE in regions of the dog population at level 5 was lower than that at level 4, which probably because the areas with the largest dog population have fewer yak or sheep populations (Fig 3). For AE, the average prevalence in regions of dog population (from level 1 to 4) are 0.49‰, 1.10‰, 2.56‰, and 4.71‰, illustrating the dog population risks AE prevalence, which is consistent with previous studies [22, 25, 27]. However, it is worth mentioning that the average prevalence of AE in regions of the dog population at level 5 (1.70‰) was lower than that at level 3 (2.56‰), which is probably due to the small distribution of yak and sheep population in this area. Regions with higher dog populations are high-risk areas for AE, but AE is also affected by other biological factors such as the distribution of yak and sheep populations, which may due to areas with higher yak and sheep populations are more prone to wild animals and rats. In conclusion, risk detection manifests that regions with larger animal populations are riskier areas for infecting with CE and AE, which further supports our conclusion.

It should be noted that this study has several limitations. Firstly, though the wildlife cycle is important in the transmission of AE as well [27], the populations of those wild animals (e.g. rats and foxes) in Tibet are not available at present, making it impossible to consider this part at this moment. Secondly, the uncertainty of stray dogs is a major challenge in the control of echinococcosis. For instance, yaks or sheep are commonly home-slaughtered or killed at a village open slaughter site due to the lack of slaughterhouses in Tibet, attracting stray dogs congregating around slaughter sites to ingest the cyst infected offal, leading to their infection with the diseases and thereby getting humans infected. However, the dog population used in this study was constricted to domestic dogs, while stray dogs are not included due to the lack of data. Thirdly, since echinococcosis is a chronic disease, several years may elapse between infection and the onset of clinical symptoms, thusly causing significant differences in the prevalence of echinococcosis among different age group[14], for example, the highest CE prevalence between 0 ~ 10 occurred in Pulan County of Ali Region, with the value of 2.85%, but the overall prevalence was just 1.56% in the area. It should be noted that the prevalence in children (aged 0 ~ 10) may be better to associate the impact of risky factors (geographical, meteorological, biological, and socio-economic) on prevalence over the past decade, but we used the overall prevalence of echinococcosis in the present study, which may increase the uncertainty of the results.

In future work, we will strengthen the collection and management of data on animal hosts (especially on wild animals and stray dogs), and further conduct detailed studies on echinococcosis considering the differences of age groups, which is of great significance to the epidemic research of infectious diseases.

## Supporting information

S1 Table The q-statistic of related spatial covariates based on the Geo-detector. (DOCX)

S2 Table The P-value of q-statistic calculated based on the Geo-detector. (DOCX)

S3 Table The interactive q-statistic values of related spatial covariates for CE. (DOCX)

S4 Table The interactive q-statistic values of related spatial covariates for AE. (DOCX)

S5 Table Risk detector of biological factors. (DOCX)
