# Supplementary material for: Geographical Detector-based influence factors analysis for Echinococcosis prevalence in Tibet, China
Source: PLoS Negl Trop Dis. 2021 Jul 12;15(7):e0009547. doi: 10.1371/journal.pntd.0009547 (PMC8297938; doi:10.1371/journal.pntd.0009547)
Supplement: S1 Table — (DOCX) [file pntd.0009547.s001.docx]

**S1 Table. The q-statistic of related spatial covariates based on the Geo-detector**

| **Indicator** | **A** | **B** | **C** | **D** | **E** | **F** | **G** | **H** | **I** | **J** | **K** | **L** |  |
| --- | --- | --- | --- | --- | --- | --- | --- | --- | --- | --- | --- | --- | --- |
| **CE** | 0.26 | 0.16 | 0.07 | 0.05 | 0.05 | 0.06 | 0.07 | 0.07 | 0.06 | 0.04 | 0.04 | 0.08 | |
| **AE** | 0.22 | 0.06 | 0.37 | 0.05 | 0.09 | 0.02 | 0.02 | 0.01 | 0.06 | 0.01 | 0.01 | 0.02 | |

Note: A = Yak population, B = Sheep population, C = Dog population, D = Population, E = GDP, F = Minimum temperature, G = Maximum temperature, H = Relative humidity, I = Precipitation, J = Terrain, K = Land use type, L = NDVI.
