# Supplementary material for: Geographical Detector-based influence factors analysis for Echinococcosis prevalence in Tibet, China
Source: PLoS Negl Trop Dis. 2021 Jul 12;15(7):e0009547. doi: 10.1371/journal.pntd.0009547 (PMC8297938; doi:10.1371/journal.pntd.0009547)
Supplement: S2 Table — (DOCX) [file pntd.0009547.s002.docx]

**S2 Table. The P-value of q-statistic calculated based on the Geo-detector**

| **P-value** | **A** | **B** | **C** | **D** | **E** | **F** | **G** | **H** | **I** | **J** | **K** | **L** |
| --- | --- | --- | --- | --- | --- | --- | --- | --- | --- | --- | --- | --- |
| **CE** | 2.12e-10 | 8.40e-11 | 8.37e-11 | 1.88e-10 | 1.18e-10 | 1.60e-10 | 7.21e-11 | 1.81e-10 | 3.06e-10 | 2.51e-9 | 1.12e-9 | 4.14e-10 |
| **AE** | 5.77e-10 | 1.12e-10 | 6.14e-10 | 9.28e-11 | 1.36e-10 | 6.67e-4 | 4.57e-5 | 0.017 | 5.43e-10 | 0.001 | 0.03 | 4.62e-5 |

Note: A = Yak population, B = Sheep population, C = Dog population, D = Population, E = GDP, F = Minimum temperature,

G = Maximum temperature, H = Relative humidity, I = Precipitation, J = Terrain, K = Land use type, L = NDVI.
