# Supplementary material for: Geographical Detector-based influence factors analysis for Echinococcosis prevalence in Tibet, China
Source: PLoS Negl Trop Dis. 2021 Jul 12;15(7):e0009547. doi: 10.1371/journal.pntd.0009547 (PMC8297938; doi:10.1371/journal.pntd.0009547)
Supplement: S4 Table — (DOCX) [file pntd.0009547.s004.docx]

**S4 Table. The interactive q-statistic values of related spatial covariates for AE**

| **Interaction** | **A** | **B** | **C** | **D** | **E** | **F** | **G** | **H** | **I** | **J** | **K** | **L** |
| --- | --- | --- | --- | --- | --- | --- | --- | --- | --- | --- | --- | --- |
| **A** | - | 0.52 | 0.55 | 0.31 | 0.4 | 0.35 | 0.36 | 0.36 | 0.45 | 0.29 | 0.3 | 0.37 |
| **B** | 0.52 | - | 0.58 | 0.38 | 0.3 | 0.28 | 0.24 | 0.24 | 0.14 | 0.08 | 0.09 | 0.14 |
| **C** | 0.55 | 0.58 | - | 0.57 | 0.57 | 0.47 | 0.47 | 0.49 | 0.5 | 0.43 | 0.44 | 0.49 |
| **D** | 0.31 | 0.38 | 0.57 | - | 0.15 | 0.11 | 0.13 | 0.2 | 0.21 | 0.07 | 0.09 | 0.11 |
| **E** | 0.4 | 0.3 | 0.57 | 0.15 | - | 0.16 | 0.17 | 0.23 | 0.18 | 0.12 | 0.12 | 0.14 |
| **F** | 0.35 | 0.28 | 0.47 | 0.11 | 0.16 | - | 0.03 | 0.06 | 0.11 | 0.03 | 0.03 | 0.04 |
| **G** | 0.36 | 0.24 | 0.47 | 0.13 | 0.17 | 0.03 | - | 0.08 | 0.13 | 0.03 | 0.04 | 0.04 |
| **H** | 0.36 | 0.24 | 0.49 | 0.2 | 0.23 | 0.06 | 0.08 | - | 0.12 | 0.03 | 0.02 | 0.05 |
| **I** | 0.45 | 0.14 | 0.5 | 0.21 | 0.18 | 0.11 | 0.13 | 0.12 | - | 0.09 | 0.09 | 0.11 |
| **J** | 0.29 | 0.08 | 0.43 | 0.07 | 0.12 | 0.03 | 0.03 | 0.03 | 0.09 | - | 0.02 | 0.03 |
| **K** | 0.3 | 0.09 | 0.44 | 0.09 | 0.12 | 0.03 | 0.04 | 0.02 | 0.09 | 0.02 | - | 0.03 |
| **L** | 0.37 | 0.14 | 0.49 | 0.11 | 0.14 | 0.04 | 0.04 | 0.05 | 0.11 | 0.03 | 0.03 | - |

Note: The values in the table is represented by color scale. The larger the value, the darker the color.
