# Supplementary material for: Geographical Detector-based influence factors analysis for Echinococcosis prevalence in Tibet, China
Source: PLoS Negl Trop Dis. 2021 Jul 12;15(7):e0009547. doi: 10.1371/journal.pntd.0009547 (PMC8297938; doi:10.1371/journal.pntd.0009547)
Supplement: S5 Table — (DOCX) [file pntd.0009547.s005.docx]

**S5 Table. Risk detector of biological factors.**

| **CE Level** | **Yak population** | **Sheep population** | **Dog population** |
| --- | --- | --- | --- |
| 1 | 1.80% | 1.24% | 1.38% |
| 2 | 1.24% | 2.22% | 1.49% |
| 3 | 1.61% | 1.48% | 1.75% |
| 4 | 2.25% | 2.58% | 2.29% |
| 5 | 3.33% | 1.64% | 1.89% |
| **AE Level** | **Yak population** | **Sheep population** | **Dog population** |
| 1 | 2.73‰ | 2.12‰ | 0.49‰ |
| 2 | 0.96‰ | 2.34‰ | 1.10‰ |
| 3 | 3.44‰ | 1.05‰ | 2.56‰ |
| 4 | 1.99‰ | 3.21‰ | 4.71‰ |
| 5 | 3.63‰ | 2.08‰ | 1.70‰ |
